# A Vision-Based Method for Detecting the Position of Stacked Goods in Automated Storage and Retrieval Systems

**DOI:** 10.3390/s25082623

**Published:** 2025-04-21

**Authors:** Chuanjun Chen, Junjie Liu, Haonan Yin, Biqing Huang

**Affiliations:** 1 Department of Automation, Tsinghua University, Beijing 100084, China or chenchuanjun@bzkj.cn (C.C.); yhn21@mails.tsinghua.edu.cn (H.Y.); 2 BZS (Beijing) Technology Development Co., Ltd., No.1 Jiaochangkou, Deshengmenwai, Beijing 100120, China; liusheng0@yeah.net

**Keywords:** automated storage and retrieval systems, computer vision, object detection, object segmentation

## Abstract

Automated storage and retrieval systems (AS/RS) play a crucial role in modern logistics, yet effectively monitoring cargo stacking patterns remains challenging. While computer vision and deep learning offer promising solutions, existing methods struggle to balance detection accuracy, computational efficiency, and environmental adaptability. This paper proposes a novel machine vision-based detection algorithm that integrates a pallet surface object detection network (STEGNet) with a box edge detection algorithm. STEGNet’s core innovation is the Efficient Gated Pyramid Feature Network (EG-FPN), which integrates a Gated Feature Fusion module and a Lightweight Attention Mechanism to optimize feature extraction and fusion. In addition, we introduce a geometric constraint method for box edge detection and employ a Perspective-n-Point (PnP)-based 2D-to-3D transformation approach for precise pose estimation. Experimental results show that STEGNet achieves 93.49% mAP on our proposed GY Warehouse Box View 4-Dimension (GY-WSBW-4D) dataset and 83.2% mAP on the WSGID-B dataset, surpassing existing benchmarks. The lightweight variant maintains competitive accuracy while reducing the model size by 34% and increasing the inference speed by 68%. In practical applications, the system achieves pose estimation with a Mean Absolute Error within 4 cm and a Rotation Angle Error below 2°, demonstrating robust performance in complex warehouse environments. This research provides a reliable solution for automated cargo stack monitoring in modern logistics systems.

## 1. Introduction

With the rapid development of the modern logistics industry, automated storage and retrieval systems (AS/RS) have emerged as crucial infrastructure in logistics systems, playing a vital role in improving space utilization and operational efficiency. However, monitoring the stability and safety of stacked goods in these systems remains challenging during actual operations. Traditional manual inspection methods fail to meet the dual efficiency and accuracy requirements in large-scale, high-intensity warehouse operations. Although automated detection equipment, such as photoelectric sensors, has been deployed to some extent, its practical effectiveness remains limited due to the necessity for multiple detection units and insufficient precision.

In recent years, computer vision and deep learning technologies have demonstrated significant potential in warehouse management systems, particularly in automated perception and digital representation of cargo status. However, a critical gap remains in specialized vision-based solutions for AS/RS environments. While some vision systems have been adapted for warehouse applications, they encounter challenges in balancing computational efficiency with detection accuracy, a crucial requirement for real-time monitoring of stacked goods. Current approaches [1,2] either rely on semantic segmentation, which suffers from high computational overhead and limited generalization in dynamic warehouse environments, or employ basic object detection [3,4] methods that struggle with accurate 3D spatial information acquisition.

In the context of AS/RS, these dynamic warehouse environments present multifaceted challenges that we characterize as “complex environments”. Such complexity manifests in three primary aspects: (1) variable lighting conditions, including shadows, uneven illumination, and energy-saving low-light periods; (2) complex backgrounds with structural elements and conveyor systems creating occlusion patterns; and (3) dynamic elements from personnel and equipment movement. These conditions represent practical operational realities rather than idealized laboratory settings. Conventional approaches would require costly environmental modifications or additional equipment to standardize these conditions, contradicting the economic efficiency principles of modern warehouse management.

**Figure 1:**
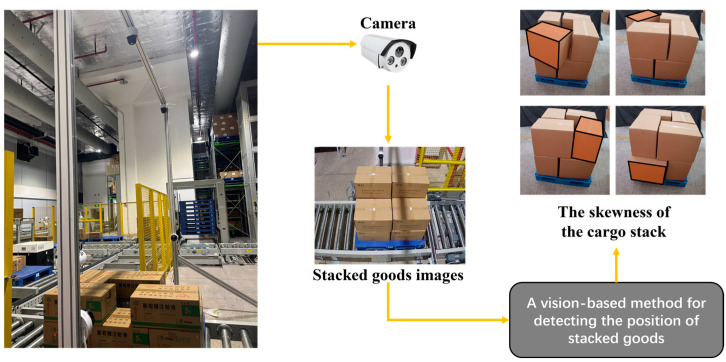
The machine vision-based system for cargo stack skewness anomaly detection. The cargo boxes shown in the figure are used for storing medications, and the text on the surface displays the medication names and specification information.

Additionally, existing detection networks often lack specialized architectures for handling the unique characteristics of warehouse scenarios, such as varying lighting conditions and complex spatial arrangements of stacked goods. These limitations underscore the necessity for an integrated approach that combines efficient feature extraction networks with geometric constraints and precise 3D pose estimation capabilities. To implement such an integrated approach, we design a vision-based detection framework, as illustrated in Figure 1. The system employs multiple cameras mounted above the conveyor line to capture comprehensive views of stacked cargo in AS/RS environments. The captured multi-angle images are processed through our proposed vision-based method, which combines deep learning-based detection with geometric analysis to identify stacking anomalies. As shown on the right side of Figure 1, the system can effectively detect various types of cargo skewness where boxes extend beyond pallet boundaries, demonstrating its capability in real-world warehouse scenarios. This framework provides a foundation for automated stack stability monitoring while addressing the existing approaches’ challenges.

To address these limitations, this research presents the following key contributions:

1. Development of STEGNet architecture and its lightweight variant MixFEGNet: We have engineered STEGNet based on STCNet, innovatively integrating fusion attention mechanisms (GFF + LAM) with the EG-FPN module. STEGNet achieves 93.49% mAP on the GY-WSBW-4D dataset and 83.2% mAP on WSGID-B, substantially outperforming baseline models. This innovation effectively addresses existing deep learning models’ inadequate feature extraction capabilities in complex warehouse environments.
2. Proposal of a hybrid methodology integrating object detection networks with edge detection algorithms: Through Region of Interest (ROI)-guided analysis and geometric constraints, this approach significantly mitigates environmental interference, achieving pose estimation with a Mean Absolute Error within 4 cm, Root Mean Square Error below 6 cm, and Rotation Angle Error under 2°. This strategy overcomes the limitations of conventional edge detection methods in complex backgrounds, enabling consistent system performance in variable warehouse conditions.
3. Implementation of Perspective-n-Point (PnP) algorithm-based 3D pose estimation from 2D imagery: We have constructed the GY-WSBW-4D and WSBW-Err datasets, with STCNet achieving an 80.8% success rate and MixFEGNet attaining a 76.3% success rate on the WSBW-Err dataset. Notably, MixFEGNet’s model size is only 66% of the baseline model and delivers 68% faster inference speed while maximally preserving accuracy, characteristics particularly advantageous for deployment in resource-constrained operational warehouse environments.

This research not only surmounts the limitations of traditional photoelectric detection equipment but also resolves reliability issues in complex environments, providing robust support for automated storage and retrieval systems. In contrast to existing studies, our methodology emphasizes lightweight design and real-time performance while maintaining high accuracy, factors crucial for practical warehouse applications.

## 2. Related Work

### 2.1. Object Detection Algorithms

Object detection networks form the foundation of our research algorithm, which can be categorized into feature-based and CNN-based visual detection algorithms.

Feature-based visual detection algorithms employ a three-step process (region proposal selection, feature extraction, and classifier recognition) using manually defined features like SIFT [5], HOG [6], and SURF [7]. Neural network-based detection algorithms are divided into two architectural approaches: single-stage and two-stage. Single-stage algorithms process images directly through regression-based recognition, exemplified by SSD [8], YOLO [9,10,11,12], and DETR [13,14,15] series. Two-stage algorithms utilize sequential modules, first generating region proposals, followed by classification and regression, with representative algorithms including RCNN [16], FastRCNN [17], FasterRCNN [18], and CascadeRCNN [19].

CNN-based object detection algorithms typically comprise backbone networks, neck modules, and detection heads. Backbone networks extract multi-level features and include lightweight networks (MobileNet [20], EfficientNet [21]), deep networks (ResNet [22], ResNeXt [23]), and Transformer-based architectures (Swin Transformer [24], ViT [25]). The neck module, positioned between the backbone and detection heads, performs feature fusion across different scales through algorithms like FPN [26], PANet [27], and BiFPN [28] to enhance the detection of various-sized objects. Detection heads generate results (categories, locations, confidence scores). They are categorized as single-stage (YOLO [9,10,11,12], SSD [8], DETR [13,14,15]) or two-stage (FasterRCNN [18], MaskRCNN [16], CascadeRCNN [19]), performing both classification and bounding box regression functions.

Despite their capabilities, existing detection algorithms face limitations in warehouse settings: feature-based methods struggle with uniform cargo appearances, while deep learning approaches often sacrifice efficiency for accuracy. Conventional networks require substantial computational resources and lack specialized mechanisms for warehouse-specific challenges such as varying lighting and complex stacking arrangements. Our research addresses these gaps by integrating object detection with edge detection algorithms, creating a hybrid approach that effectively balances computational efficiency with detection accuracy while maintaining robustness in complex warehouse environments.

### 2.2. Pose Detection Algorithms

Machine vision-based cargo pose estimation methods can be categorized into feature matching and deep learning approaches. They are further divided based on whether they utilize two-dimensional or three-dimensional image information.

In 2D methods, feature matching approaches include feature point-based algorithms like PnP [29], SIFT [29], and SURF [30], which detect distinctive local features. Edge detection methods such as Hough transform [31], FAST, and ORB [32] focus on structural boundaries. Deep learning contributions include CNN-based segmentation architectures (FCN [33], U-Net [34], SegNet [35], PSPNet [36], DeepLab [37]) and Transformer-based models (ViT [25], Segmenter [38], SETR [39], Swin-UNet [40], SAM [41]). Image descriptor methods like SuperPoint [42] and DELF [43] provide robust feature detection capabilities.

For 3D methods, feature matching primarily employs point cloud techniques including ICP [44,45] and FPFH [46], alongside shape-based methods like SAC-IA [47]. Deep learning approaches include 3D descriptors through D2-Net [48] and 3DMatch [49], specialized architectures like PointNet [50] and PointNetLK [51], CNN adaptations (3D U-Net [52], 3D SegNet [53], VoxelNet [54]), and Transformer architectures such as Point Transformer [55]. Multimodal approaches like Pix2Pose [56] leverage multiple data representations for robust pose estimation.

Feature matching in 2D determines object pose by comparing corresponding features between the target and template images. At the same time, 3D approaches typically employ point cloud matching through rigid transformations to convert object point clouds to target point clouds. The core concept of ICP [44,45] involves iteratively transforming to minimize the Euclidean distance between corresponding points.

With deep learning advancements, neural network-based pose estimation has emerged, falling into two categories: image descriptor-based approaches for extracting key points and segmentation-based methods for partitioning images into regions with specific semantic properties. Unlike simpler visual tasks, image segmentation requires pixel-wise classification, creating higher training difficulty and computational demands. The field continues evolving through CNN-based [52,53,54] and Transformer-based [55] innovations.

Current pose estimation methods present several challenges in warehouse applications: 2D approaches suffer from lighting sensitivity and struggle with repetitive cargo patterns; deep learning methods impose excessive computational demands; and 3D techniques require specialized hardware, increasing system cost. Most approaches also lack integration between detection and pose estimation. Our methodology overcomes these limitations by combining efficient detection with geometric constraints, enabling accurate pose estimation from monocular images while maintaining computational efficiency.

### 2.3. Application of Machine Vision in Warehouses

Machine vision is a prominent research direction in automated storage and retrieval systems, particularly in warehouse cargo recognition. He et al. [57] investigated machine vision-based cargo classification methods in warehousing, developing solutions capable of simultaneously identifying warehouse cargo with diverse shapes and colors while addressing the inadequate performance of static visual image processing solutions in optimizing recognition speed and classification accuracy. Daios et al. [58] conducted a comprehensive literature review of visual methods for automated warehouse inventory, encompassing technologies such as OCR, 3D modeling, and drone imaging. Yoneyama et al. [59] investigated visual depth estimation in automated warehouse scenarios to enhance the operational precision of warehouse robots. To the best of our knowledge, we are the first to implement a machine vision-based system for detecting anomalies related to cargo stack skewness.

Analysis of existing research reveals critical limitations: (1) current detection methods struggle to balance efficiency with accuracy in warehouse environments; (2) feature-matching techniques show limited robustness for lighting variations; (3) deep learning models, while accurate, are often too resource-intensive for real-time deployment; and (4) insufficient attention has been given to automated monitoring of stacking stability. Our work addresses these gaps through a specialized framework that combines object detection networks with geometric edge detection algorithms, creating a hybrid approach that optimizes the trade-off between computational overhead and detection precision for efficient cargo stack monitoring.

## 3. Methodology

### 3.1. Overall Framework

**Figure 2:**
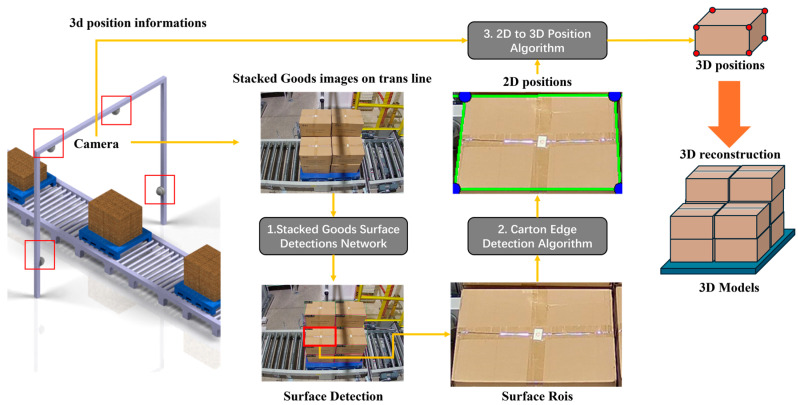
Pipeline architecture of the proposed 2D image-based cargo stack pattern detection algorithm for automated storage and retrieval systems (AS/RS), illustrating the three main processing components: stack surface detection, box edge detection, and pose estimation. The cargo boxes shown in the figure are used for storing medications, and the text on the surface displays the medication names and specification information.

As shown in Figure 2, our proposed 2D image-based cargo stack pattern detection algorithm for AS/RS consists of three main components: stack surface detection, box edge detection, and pose estimation. The details of their implementation and the pipeline are outlined as follows:

- Stack Surface Detection: multi-angle stack view images captured from four distinct perspectives are input into the stack surface detection network to identify each box surface as Regions of Interest (ROIs) for subsequent processing.
- Box Edge Detection: edge detection is performed by traversing the ROIs of each box surface from various angles to locate the actual edges and corner points in two-dimensional coordinates.
- Pose Estimation: the two-dimensional coordinates of box surface corner points, along with the camera intrinsic matrix, distortion coefficients, and box shape matrix, are provided to the pose estimation algorithm to calculate the boxes’ three-dimensional coordinates.

Based on the obtained 3D coordinates, we calculate the cargo stacking pattern and determine whether the boxes are tilted or extending beyond the pallet boundaries according to preset thresholds, thereby ensuring the safety of cargo transportation.

### 3.2. Stacked Goods Surface Detection Network

**Figure 3:**
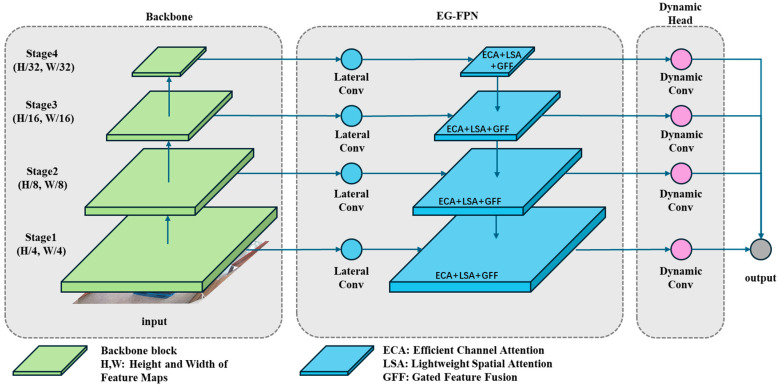
STEGNet overall structure.

Our proposed object detection network, Swin Transformer Enhanced Gated Network (STEGNet), is developed based on STCNet [60], which has demonstrated excellent performance in box surface defect detection on stacked pallets. While STCNet effectively leverages the Swin Transformer backbone, Feature Pyramid Network (FPN) neck, and cascaded detector head, we identified opportunities to enhance its architecture further for box inspection scenarios. To reduce parameter scale and improve prediction efficiency, we optimized the network’s neck and head components by designing an Efficient Gated Pyramid Feature Network (EG-FPN) that integrates Gated Feature Fusion (GFF) and Lightweight Attention Mechanism (LAM) modules. The GFF selectively combines multi-scale features through learnable gates, addressing the information flow bottlenecks observed in the original neck structure. At the same time, LAM captures long-range dependencies with reduced computational complexity. Additionally, we selected Dynamic Head to replace the traditional Cascade Head, enabling instance-aware feature aggregation that better accommodates the varying scales and appearances of surface defects commonly found on stacked box surfaces. Our analysis of real-world box inspection challenges motivated these architectural choices, where computational efficiency and detection accuracy for diverse defect types are critical requirements. The overall architecture is illustrated in Figure 3.

Furthermore, to enhance the model prediction speed while reducing the model size and complexity, we experimented with MixFormer Enhanced Gated Network (MixFEGNet), which uses MixFormer as its backbone. This model achieved a 33% reduction in model size and nearly doubled the inference speed while only experiencing a 1–3% decrease in mAP, meeting our task’s accuracy requirements.

#### 3.2.1. Feature Extractor

**Figure 4:**
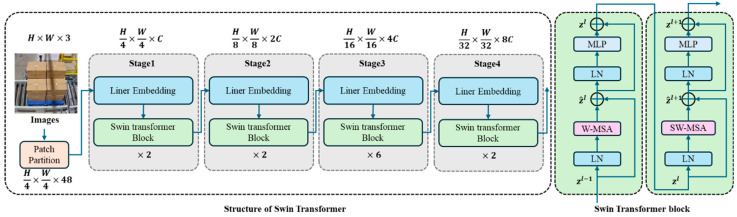
Structure of Swin Transformer and Swin Transformer block.

The backbone network of our proposed method employs two advanced architectures: Swin Transformer and MixFormer [61]. The Swin Transformer, which won the best paper award at ICCV 2021, introduces a hierarchical architecture that computes self-attention within local windows. For an input feature map of size H × W, it first partitions the image into non-overlapping patches and performs window-based multi-head self-attention (W-MSA) within each local window. Shifted window partitioning (SW-MSA) is implemented in successive blocks to enable cross-window information exchange. This design significantly reduces computational complexity while maintaining feature extraction capability. Figure 4 shows the structure of the Swin Transformer.

**Figure 5:**
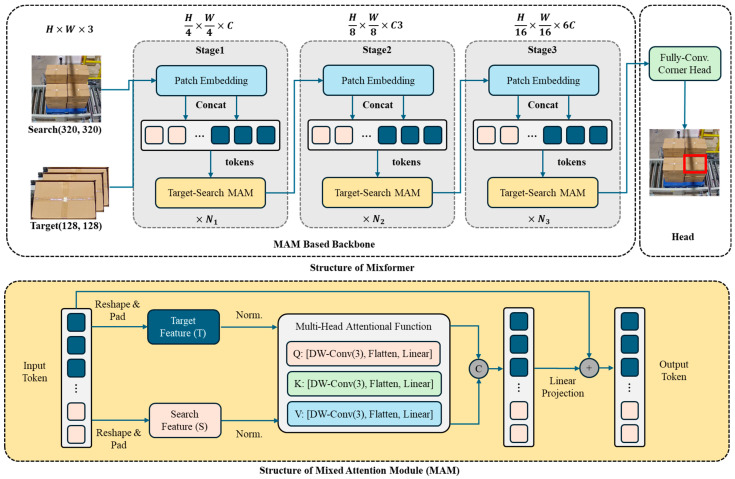
Structure of MixFormer.

The MixFormer architecture complements the Transformer-based approach by incorporating both CNN and Transformer characteristics. It employs a hybrid structure (Mixed Attention Module, MAM), where token and channel mixing are performed separately. The token mixing module captures spatial dependencies through lightweight depth-wise convolutions, while the channel mixing module utilizes feed-forward networks to process channel-wise information. This design effectively combines the local feature extraction capabilities of CNNs with the global modeling abilities of Transformers while maintaining computational efficiency. Our proposed feature fusion network then processes the multi-scale feature maps generated by both extractors for subsequent detection tasks. Figure 5 shows the structure of the Mixformer.

#### 3.2.2. EG-FPN

**Figure 6:**
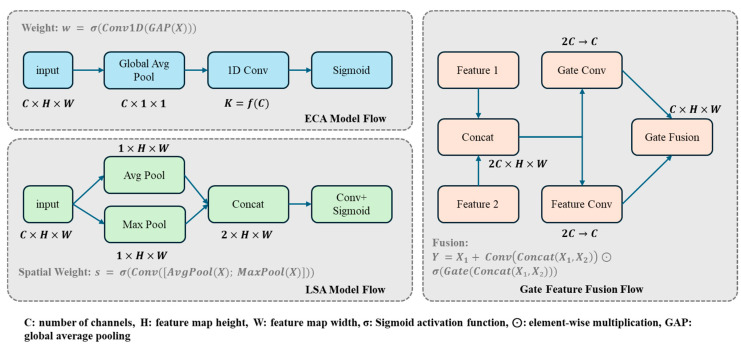
Structure of EG-FPN.

To address the balance between efficiency and expressiveness in multi-scale feature extraction and fusion for object detection, we propose a lightweight feature pyramid network structure—Efficient Gated Pyramid Feature Network (EG-FPN). This network innovatively incorporates efficient attention mechanisms and gated feature fusion modules, significantly reducing computational complexity while enhancing multi-scale feature modeling capabilities. The Structure of EG-FPN is shown in Figure 6.

In EG-FPN, we designed an efficient Gated Feature Fusion (GFF) module. Given multi-scale feature maps $C_{2},C_{3},C_{4},C_{5}$ from the backbone network, the GFF module performs channel dimensionality reduction and feature mapping through group convolution, generating lateral features $P_{2},P_{3},P_{4},P_{5}$. For feature maps $x_{l}\in R^{C\times H\times W}$ and $x_{l+1}\in R^{C\times H/2\times W/2}$ from adjacent levels, the GFF module dynamically controls the feature fusion process through a gating mechanism:

(1) $$F_{fuse}=G⊙F_{conv}+x_{l}$$

(2) $$G=\sigma \left(W_{gate}\cdot Concat\left(x_{l},Upsample\left(x_{l+1}\right)\right)\right)$$

where G represents the fusion gating weight, $W_{gate}$ is the learnable weight matrix, and $F_{conv}$ denotes the fused features obtained through group convolution and lightweight convolution. The proposed gating mechanism enables the network to adaptively control information flow between different feature levels, enhancing the model’s ability to capture multi-scale features.

To further enhance feature representation capabilities, we introduced a Lightweight Attention Mechanism (LAM) into the network. LAM comprises Efficient Channel Attention (ECA) and Lightweight Spatial Attention (LSA). The efficient channel attention branch captures inter-channel relationships through 1D convolution while reducing parameter count:

(3) $$M_{c}\left(X\right)=\sigma \left(Conv_{1D}\left(GlobalPool\left(X\right)\right)\right)$$

where $GlobalPool\left(X\right)$ represents the global average pooling operation. By using 1D convolution, the channel attention mechanism efficiently models interdependencies with minimal computational overhead.

The lightweight spatial attention branch reduces the computational cost of spatial modeling through depth-separable convolution:

(4) $$M_{s}\left(X\right)=\sigma \left(Conv_{7\times 7}\left(Concat\left[MaxPool_{c}\left(X\right),AvgPool_{c}\left(X\right)\right]\right)\right)$$

where $MaxPool_{c}$ and $AvgPool_{c}$ represent max pooling and average pooling operations along the channel dimension, respectively. Through depth-separable convolution, the spatial attention formulation effectively captures positional information while maintaining computational efficiency.

During feature fusion, we further optimized the feature transmission path. For features at layer l, the enhanced representation is:

(5) $$F_{l}=x_{l}+\alpha M_{c}\left(x_{l}\right)⊙x_{l}+\beta M_{s}\left(x_{l}\right)⊙x_{l}$$

where α and β are learnable parameters balancing contributions from different attention modules, and ⊙ denotes element-wise multiplication. With learnable parameters α and β, the network can focus on the most informative features from both channel and spatial dimensions.

Subsequently, feature fusion is achieved through the gating fusion mechanism combined with upsampling:

(6) $$Y_{l}=GFF\left(F_{l},Upsample\left(Y_{l+1}\right)\right)$$

The recursive fusion operation progressively integrates high-level semantic information with low-level spatial details, creating feature maps with rich contextual information.

The overall structure of EG-FPN implements an efficient framework for feature extraction and fusion. The bottom–up pathway gradually extracts hierarchical semantic features through group convolution, while the top–down pathway enhances feature representation through gated fusion and lightweight attention. This network significantly reduces computational cost while maintaining high-precision object detection capabilities, making it suitable for resource-constrained scenarios such as real-time detection tasks and embedded device deployment.

#### 3.2.3. Dynamic Cascade Output Head

In the network’s ROI output head, using only a threshold of IoU = 0.5 to determine whether predicted bounding boxes match ground truth leads to lower detection accuracy. The network may output more false-positive prediction boxes, which more severely impact post-processing algorithms than missed detections. Furthermore, it is challenging for a single detection head to perform well across all IoU threshold levels. Therefore, we employ Dynamic Head to improve detection accuracy, particularly for target regions with variable sizes and ambiguous features.

Dynamic Head efficiently connects with the EG-FPN neck by introducing multi-scale, spatial, and task attention mechanisms. After multi-stage regression, each ROI is optimized based on dynamically adjusted attention weights rather than fixed IoU threshold matching. Under this multi-task-aware mechanism, Dynamic Head better adapts to different feature levels and regions, performing fine-grained regression on higher-quality prediction boxes. Through our implementation, compared to the baseline model STCNet, the Dynamic Head introduced in STEGNet effectively improves bounding box regression accuracy and significantly reduces false-positive predictions while demonstrating excellent performance in detecting ambiguous and complex targets.

### 3.3. Box Edge Detection Algorithm

**Figure 7:**
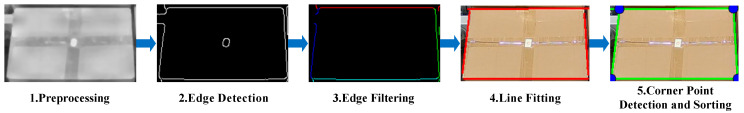
Steps of the Box Edge Detection Algorithm. The red, cyan, blue, and green edges in 3 represent the top, left, bottom, and right edges, respectively; the red line segment in 4 represents the result of Line Fitting; the green straight line in 5 is the result of extending the line segment, and the blue circle represents the corner point.

As shown in Figure 7, the box edge detection algorithm obtains the actual position information of boxes within target regions on the box surface. The algorithm comprises image preprocessing, edge detection, edge filtering, line fitting, and corner point detection and sorting, with each step detailed in the following subsections.

#### 3.3.1. Image Preprocessing

**Figure 8:**
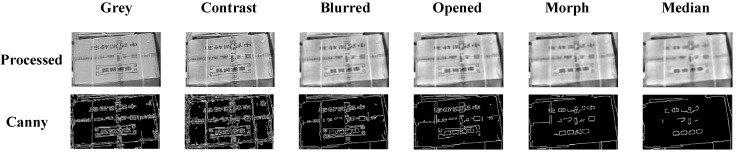
Each step result of image preprocessing. The cargo boxes shown in the figure are used for storing medications, and the text on the surface displays the medication names and specification information.

Image preprocessing is a crucial step for subsequent edge detection and line extraction. In this paper, we apply grayscale conversion, contrast enhancement, Gaussian filtering, morphological processing, and median filtering to preprocess images, reducing image noise and improving the robustness of subsequent processing. Figure 8 demonstrates the processing effects.

Grayscale Conversion: Color images contain RGB information across three channels, while in box corner detection tasks, we primarily focus on structural features rather than color information. Therefore, we first convert RGB images to grayscale images. The RGB-to-grayscale conversion uses a weighted average method:

(7) Gray=0.299R+0.587G+0.114B

where R, G, and B represent the red, green, and blue channel values of pixels, respectively. The weight coefficients are determined based on human eye sensitivity to different colors. Grayscale conversion transforms three-channel images into single-channel ones, reducing data volume while preserving the main structural information.

Adaptive Contrast Enhancement: We apply the CLAHE algorithm to grayscale images to enhance local image contrast. For each local region in the image, histogram equalization can be expressed as:

(8) $$PDF\left(i\right)=\frac{n_{i}}{N},i\in \left[0,L-1\right]$$

(9) $$CDF\left(i\right)=\sum_{j=0}^{i}PDF\left(j\right)$$

(10) $$f\left(v\right)=v_{min}+\left(v_{max}-v_{min}\right)\cdot CDF\left(v\right)$$

where $n_{i}$ represents the number of pixels at gray level i, N denotes the total number of pixels in the region, L is the number of gray levels, and $v_{min}$ and $v_{max}$ represent the target grayscale range. CLAHE redistributes pixel intensities to enhance local contrast while limiting noise amplification through histogram clipping.

Gaussian Filtering: To suppress image noise, we apply Gaussian filtering to the grayscale images. The Gaussian filter is a linear smoothing filter whose weight coefficients follow a two-dimensional Gaussian distribution:

(11) $$G\left(x,y\right)=\frac{1}{2\pi \sigma^{2}}\cdot exp\left(-\frac{x^{2}+y^{2}}{2\sigma^{2}}\right)$$

where (x,y) represents the pixel position relative to the kernel center, and x is the standard deviation of the Gaussian distribution. Following the Gaussian distribution, the filter assigns higher weights to central pixels and gradually decreasing weights to peripheral pixels, creating a balanced smoothing effect.

In practical applications, convolution operations are performed on images using discrete Gaussian kernels:

(12) $$I^{\prime }\left(i,j\right)=\sum I\left(i+m,j+n\right)\cdot G\left(m,n\right)$$

where $I^{\prime }(i,j)$ represents the pixel value at position (i,j) after filtering, I(i+m,j+n) is the corresponding pixel value in the original image, and G(m,n) is the Gaussian kernel coefficient. When applied to images, the convolution operation effectively suppresses high-frequency noise while preserving important structural features.

Morphological Processing: We apply morphological opening and closing operations to optimize image quality. Given the structuring element B and the original image I:

Opening operation:
  (13) $$I\circ B=\left(I⊖B\right)⊕B$$
Closing operation:
  (14) $$I\cdot B=\left(I⊕B\right)⊖B$$
  where ⊖ and ⊕ represent erosion and dilation operations, respectively. The opening operation removes small bright details while preserving the overall shape and size of larger objects, while the closing operation fills small holes and connects nearby objects.

Median Filtering: Finally, median filtering is applied to further suppress noise while preserving edge features. For median filtering with a window size of $\left(2w+1\right)\times \left(2w+1\right)$:

(15) $$I^{\prime }\left(x,y\right)=median\left\{I\left(x+i,y+j\right)|-w\leq i,j\leq w\right\}$$

As a non-linear technique, median filtering replaces each pixel with the median value from its neighborhood, effectively removing salt-and-pepper noise while better preserving edge integrity compared to linear filters.

#### 3.3.2. Edge Detection

Based on the preprocessed images, we employ the Canny operator to extract box edge contour information for edge detection. The Canny operator is a multi-stage edge detection algorithm with the advantages of strong noise resistance and precise localization.

Figure 8 compares original images, preprocessed images, and Canny edge detection results. Box edge contours are clearly extracted after Canny edge detection, establishing a foundation for subsequent line detection.

It should be noted that edge detection performance is influenced by factors such as image quality and lighting conditions. In practical applications, algorithm parameters may need dynamic adjustment according to specific scenarios.

#### 3.3.3. Edge Filtering

Based on edge detection results, we employ an edge filtering algorithm based on the Hough Transform [31] to filter and classify edge points by identifying major lines, providing reliable edge features for subsequent processing.

**Figure 9:**
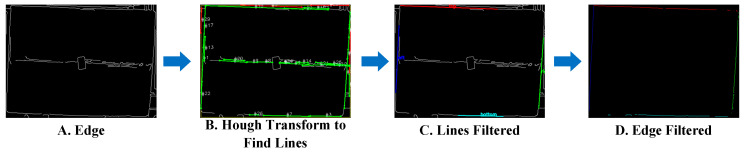
Edge filtering.

As shown in Figure 9, in real-world environments, complex background conditions behind boxes and features such as tape and printing on boxes cannot be eliminated by preprocessing steps, affecting edge detection performance. Figure 9A demonstrates the edge detection results, where tape, labels on the boxes, and the environmental background are also identified as edges. Edge filtering is used to locate the true edges of boxes.

Hough Transform Line Detection: The Hough transform maps lines from the image space to the parameter space. In polar parametric representation, any line can be represented as:

(16) $$\rho =x\cdot \cos\left(\theta \right)+y\cdot \sin\left(\theta \right)$$

where ρ represents the perpendicular distance from the line to the coordinate origin, θ represents the angle between the perpendicular and x-axis, and (x,y) represents any point on the line. The key advantage of the Hough transform lies in converting the complex problem of line detection in the image space into a peak-finding problem in the parameter space, making it computationally more tractable.

To improve computational efficiency, we employ the Probabilistic Hough Transform (PHT). The main parameters of PHT include ρ resolution (in pixels), θ resolution (in radians), minimum voting threshold, minimum line segment length, and maximum line segment gap.

Figure 9B shows the results of applying the Hough transform for line detection and filtering. Numerous candidate line segments are detected due to background and box surface texture effects. In subsequent steps, we apply specific rules to select the segments most likely to represent box edges.

Edge Point Filtering Principle: Given an edge image E(x,y) and image dimensions (H,W), calculate the distance from each edge point (x,y) to the major lines and filter through distance thresholds:

(17) $$D\left(x,y\right)=\frac{\left|ax+by+c\right|}{\sqrt{a^{2}+b^{2}}}$$

where (a,b,c) represents line parameters, and D(x,y) denotes the distance from point to line. This distance calculation bridges the line detection in Figure 9B to the edge classification in Figure 9C by assigning each edge point to its nearest line when the distance falls below a threshold. Figure 9C displays the resulting classification where edge points are successfully categorized into four distinct box boundaries (top, bottom, left, and right), effectively separating true box edges from noise generated by labels or background textures.

**Figure 10:**
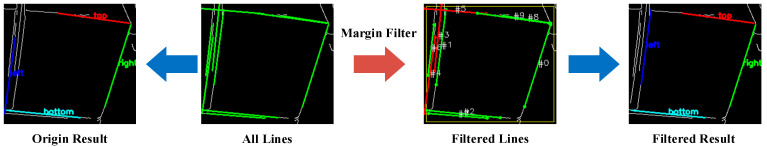
Comparison of margin filtering algorithms. In result of Filtered Lines, green lines represent retained box edges after filtering, while red lines indicate rejected line segments that were filtered out during the process.

Line Classification and Filtering: Considering the geometric characteristics of boxes and combining with Hough transform results, we classify and filter the detected lines, as shown in Figure 10.

We designed an edge line filtering strategy to exclude non-candidate box edges, edges from other box surfaces, and environmental edges from affecting filtering results. We observed that these interfering edges are often incomplete. Therefore, we designed edge thresholds to exclude candidate lines with endpoints within the edge threshold. As shown in Figure 10, without edge filtering, the algorithm incorrectly identifies other edges as target box edges, while with edge filtering, the algorithm finds the correct box edges.

After detecting edges and lines, we apply several filtering steps to enhance box edge detection. First, angle normalization standardizes line orientation. Direction classification then categorizes lines as vertical or horizontal based on an angle threshold of 45°. Position grouping assigns vertical lines to left/right regions and horizontal lines to top/bottom regions. We implement an edge proximity threshold mechanism that filters out interior lines by defining a configurable threshold (default 15% of the minimum image dimension). This ensures that only lines near the image edges are considered potential box borders. We verify that horizontal lines are sufficiently close to the top or bottom edge, while vertical lines must be near the left or right edge. This enhancement reduces false-positives in complex scenes where interior lines might compete with actual box edges, with the scoring system prioritizing lines that satisfy the edge proximity criterion.

Finally, edge point assignment maps points to their nearest lines within a distance threshold of 5 pixels, and the edges are extended outward, as shown in Figure 9D. This comprehensive filtering approach effectively classifies edge points into four distinct box boundaries, significantly improving detection accuracy and efficiency while eliminating interference from non-box edges.

#### 3.3.4. Line Fitting

**Figure 11:**
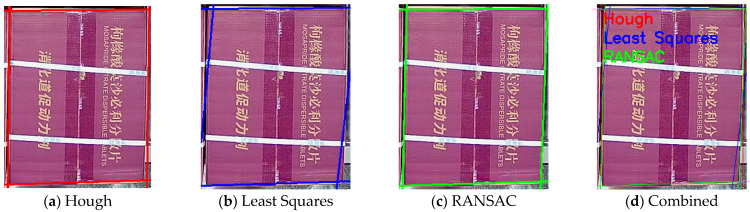
Result of line fitting. The cargo boxes shown in the figure are used for storing medications, and the text on the surface displays the medication names and specification information.

We employ the Random Sample Consensus (RANSAC) algorithm to perform line fitting for each cluster based on the clustering results of edge points. Through iterative optimization, outliers are eliminated to obtain accurate boundary line parameters. Through robust line fitting using the RANSAC algorithm, the influence of outliers is effectively eliminated, providing precise line parameters for subsequent corner localization. As shown in Figure 11, the qualitative comparison between RANSAC, the least-squares method, and the Hough transform demonstrates that RANSAC achieves superior line fitting results, particularly in handling noisy edge points.

#### 3.3.5. Corner Detection and Selection

Based on the aforementioned line detection results, this paper proposes a corner detection method utilizing line intersection computation and clustering. Through spatial geometric constraints, the corners are filtered and sorted to obtain precise corner coordinates on the upper surface of the box structure. A multi-layer selection strategy is implemented to enhance the accuracy of corner detection.

We define valid corners as points (x,y) that satisfy all of the following conditions:

- The point (x,y) lies within the region of interest: (x,y) ∈ ROI
- The angle between the two intersecting lines L_1_ and L_2_ that form the corner approximates a proper angle: $∠(L_{1},L_{2})\;\in \;[90{}^{\circ}\pm \alpha ]$
- The distance from the corner to the relevant lines falls within an acceptable range: min_dist<dist(corner,line)<max_dist

Several key parameters govern the corner detection performance in these equations. The angle tolerance α is empirically set to 10° to accommodate slight deviations from perfect perpendicularity in real-world images. The distance thresholds min_dist and max_dist are configured to filter out spurious intersection points while retaining valid corners, with values determined through experimental calibration on the target box types.

This equation bridges the line detection stage shown in Figure 7(4) to the corner identification stage in Figure 7(5) by mathematically formalizing the geometric constraints that characterize valid box corners. The detected corners undergo distance-based clustering to group closely positioned corner candidates and spatial position-based sorting using standard computational geometry techniques to achieve a consistent corner sequence representation for subsequent processing. This ordered corner sequence is essential for the box pose estimation procedure detailed in Section 1.

### 3.4. Pose Estimation

**Figure 12:**
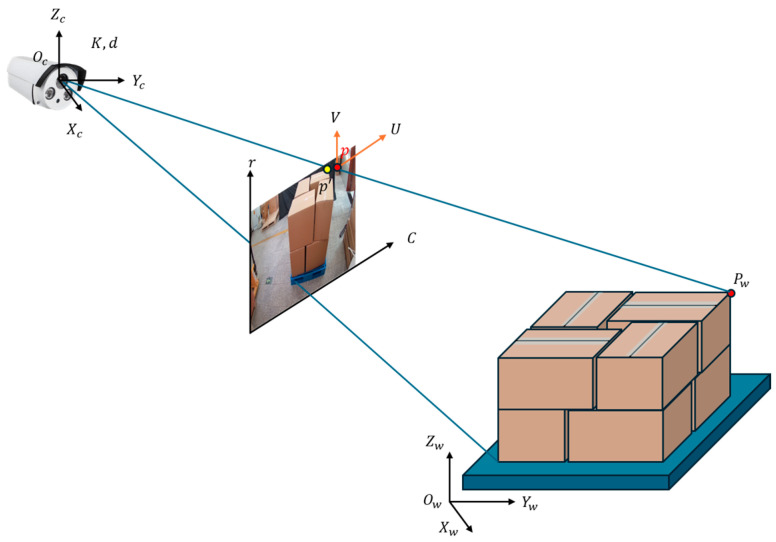
Convert 2D coordinates to 3D coordinates using the PNP algorithm.

To obtain the 3D coordinates of each box, we propose a monocular 3D pose estimation algorithm based on 2D images and geometric constraints. This algorithm combines object location information extracted from the detection network, object dimension information, and camera intrinsic parameters to solve the 3D relative pose using the PnP algorithm. As illustrated in Figure 12, the process involves coordinate transformations between multiple reference frames: the world coordinate system, camera coordinate system, 3D cargo stack coordinate system, and projection plane coordinate system. The figure demonstrates how 2D projection points are related to their corresponding 3D points through projection and distortion models. The required information and detailed procedure are presented in the following subsections.

#### 3.4.1. Camera Calibration

Camera calibration determines the geometric and optical characteristics of cameras through establishing correspondences between 3D world points and their 2D image projections. Under the pinhole camera model, the projection from 3D to 2D can be represented as:

(18) $$s\left[\begin{array}{c} u\\ v\\ 1 \end{array}\right]=K\left[R|t\right]\left[\begin{array}{c} X\\ Y\\ Z\\ 1 \end{array}\right]$$

where ${\left[u\;v\;1\right]}^{T}$ represents homogeneous image coordinates, ${\left[X\;Y\;Z\;1\right]}^{T}$ represents homogeneous world coordinates, s is the scale factor, K is the intrinsic matrix, and [R|t] is the extrinsic matrix. Serving as the mathematical foundation, this projection equation maps real-world objects onto the image plane, enabling accurate spatial measurements from images. Real cameras exhibit lens distortion, primarily radial distortion modeled as:

(19) $$x_{distorted}=x\left(1+k_{1}r^{2}+k_{2}r^{4}+k_{3}r^{6}\right)$$

(20) $$y_{distorted}=y\left(1+k_{1}r^{2}+k_{2}r^{4}+k_{3}r^{6}\right)$$

where (x, y) represents ideal undistorted image coordinates; $\left(x_{distorted},\;y_{distorted}\right)$ represents actual distorted image coordinates; $r^{2}=x^{2}+y^{2}$ is the squared distance to the optical center; and $k_{1}$, $k_{2}$, $k_{3}$ are radial distortion coefficients. These polynomial equations model how straight lines in the world appear curved in captured images, with distortion increasing radially from the center. The intrinsic matrix K contains the camera’s optical characteristics:

(21) $$K=\left[\begin{array}{ccc} f_{x} & 0 & c_{x}\\ 0 & f_{x} & c_{x}\\ 0 & 0 & 1 \end{array}\right]$$

where $f_{x}$, $f_{y}$ are focal lengths (in pixels); $c_{x}$, $c_{y}$ are principal point coordinates. Structured as a 3 × 3 matrix, K encapsulates the camera’s internal geometry and is essential for converting between pixel coordinates and normalized image coordinates. Distortion coefficients are represented as:

(22) $$d=\left[k_{1},k_{2},p_{1},p_{2},k_{3}\right]$$

where $k_{1},k_{2},k_{3}$ are radial distortion coefficients; and $p_{1},\;p_{2}$ are tangential distortion coefficients. These parameters collectively characterize the lens’ deviation from the ideal pinhole model, allowing software correction of image distortions. Our PnP algorithm exclusively utilizes the intrinsic matrix K and distortion coefficients d.

#### 3.4.2. PnP Algorithm

The PnP algorithm estimates the 3D pose of box surfaces by utilizing the four corner points detected from the previous box edge detection step. This algorithm establishes the relationship between the 2D corner points in the image plane and their corresponding 3D coordinates in the camera reference frame.

The transformation from 3D world coordinates to 2D image coordinates can be formulated as:

(23) $$p=K\left[R|T\right]P$$

where p represents 2D projection points, K is the camera intrinsic matrix, R is the rotation matrix, T is the translation vector, and P represents 3D points in world coordinates. At the core of pose estimation, this equation provides the mathematical relationship between a box’s actual position in space and its appearance in the camera image.

The algorithm minimizes the reprojection error ε using Levenberg–Marquardt optimization:

(24) $$\varepsilon =\sum ||p_{i}-proj(K,R,T,P_{i})||2$$

where proj() is the projection function that maps 3D points to 2D using the camera model. By minimizing the difference between observed corner points and their theoretical projections, the optimization approach iteratively refines the estimated pose parameters, ensuring accurate box pose determination.

Algorithm 1 provides the 3D coordinates of each box surface relative to the camera. In subsequent sections, we will demonstrate how multiple monocular views are integrated to reconstruct the complete 3D model of the stacked cargo, effectively transforming local camera coordinates into a unified world coordinate system.
**Algorithm 1:** PnP AlgorithmInput:-Box dimensions (length, width, height) -2D corner points p = {p_1_, p_2_, p_3_, p_4_} from box edge detection, where p_i_ = (u_i_, v_i_)-Camera intrinsic matrix K-distortion coefficients D
Output:
-Rotation matrix R-Translation vector T-3D coordinates of box surface corners
Step:Define the 3D model of the box surface corners in the object coordinate system:
P = {(0,0,0), (width,0,0), (0,height,0), (width,height,0)}
2.Establish 2D–3D point correspondences between detected corners and model points.3.Solve the PnP problem to obtain the rotation vector r and the translation vector T:Minimize reprojection error:ε = Σ||p_i_ − proj(K, R, T, P_i_)||^2^Use Levenberg–Marquardt optimization for this minimization.4.Convert the rotation vector r to the rotation matrix R.5.Calculate 3D coordinates of box corners in camera coordinate system:
P^cam^ = R·P + T Return R, T, P^cam^

#### 3.4.3. Multi-View Extension

To extend our single-view approach to multi-camera scenarios, we propose an integrated framework that maintains the efficiency of monocular algorithms while leveraging multi-view geometric constraints. Given a cargo stack observed by N cameras {C_1_, C_2_, …, C_n_}, we first process each view independently using our single-view pipeline, then incorporate cross-view information through a unified optimization framework.

The multi-view processing pipeline consists of three key components. First, we establish a global coordinate system through a unified camera calibration process:

(25) $$X_{w}=R_{i}Xc_{i}+t_{i}$$

where$\;X_{w}$ represents points in the world coordinate system; $Xc_{i}$ denotes camera coordinates for view i; and $\left\{R_{i},\;t_{i}\right\}$ are the rotation and translation parameters for each camera, respectively. Applying this rigid transformation establishes a common reference frame for all cameras, enabling the integration of information from multiple viewpoints.

Second, we perform cross-view detection fusion. For each detected cargo surface, we project the detection results from multiple views onto the unified coordinate system:

(26) $$X_{3D}=argmin\sum_{i}{\left||P_{i}X_{3D}-x_{i}|\right|}^{2}\;$$

where $P_{i}$ is the projection matrix for camera i, and $x_{i}$ represents the detected 2D points in view i. This optimization problem can be solved efficiently using the Direct Linear Transform (DLT) algorithm. Formulated as a least-squares problem, the equation finds the optimal 3D point that minimizes the reprojection error across all views, effectively triangulating the position from multiple observations.

Third, we refine the initial PnP-based pose estimates through multi-view constraints. For corresponding edge points $e_{i}$ detected across views, we enforce the epipolar constraint:

(27) $${e_{i}}^{T}F_{\mathrm{ij}}e_{j}=0$$

where $F_{\mathrm{ij}}$ is the fundamental matrix between views i and j. Fundamental to multi-view geometry, this constraint ensures that corresponding points across different views lie on conjugate epipolar lines, providing a powerful geometric validation mechanism for feature matches. The final pose parameters are obtained by minimizing a combined cost function:

(28) $$E=E_{\mathrm{reproj}}+\lambda E_{\mathrm{epipolar}}$$

where $E_{\mathrm{reproj}}$ represents the reprojection error from PnP estimation, $E_{\mathrm{epipolar}}$ denotes the epipolar constraint error, and λ is a weighting factor. By combining the reprojection error with epipolar constraints, the cost function balances single-view pose accuracy with multi-view geometric consistency, resulting in more robust and accurate cargo pose estimates.

The integrated framework processes multi-view data in real-time through parallel computation of single-view algorithms and efficient cross-view optimization. This approach preserves the computational efficiency of our monocular pipeline while enhancing pose estimation accuracy through geometric constraints. The system automatically supplements missing information from alternative viewpoints for occluded regions in single views, resulting in more robust pose estimation for complex stacking patterns.

The proposed multi-view extension maintains consistent performance with varying camera configurations, requiring only that adjacent cameras maintain sufficient overlap in their fields of view. This flexibility makes our method particularly suitable for practical warehouse environments where physical infrastructure may constrain camera placement.

## 4. Experiment and Results

To thoroughly evaluate the effectiveness of our proposed method, we conducted extensive experiments on the GY-WSBW-4D and WSGID-B datasets. The experiments can be categorized into three main parts: First, we present the implementation details, including camera calibration procedures, parameter settings, experimental environment setup, and evaluation metrics. Then, we perform comprehensive comparative experiments to evaluate our STEGNet against state-of-the-art models regarding detection accuracy and computational efficiency. Finally, we conduct ablation studies to validate the effectiveness of our proposed EG-FPN module by examining its impact on feature extraction and fusion capabilities.

**Figure 13:**
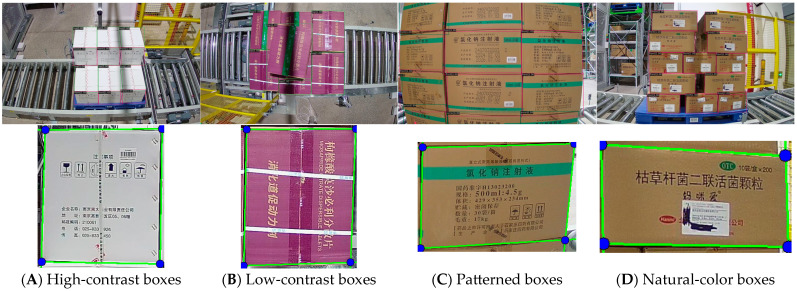
Qualitative evaluation results on the test dataset. The cargo boxes shown in the figure are used for storing medications, and the text on the surface displays the medication names and specification information.

Our qualitative results demonstrate that STEGNet achieves robust detection performance across various challenging scenarios in warehouse environments. It effectively identifies cargo surfaces and accurately estimates their 3D poses. As shown in Figure 13, our model successfully detects cargo stacks under varying lighting conditions, viewing angles, and stacking patterns.

### 4.1. Dataset

**Figure 14:**
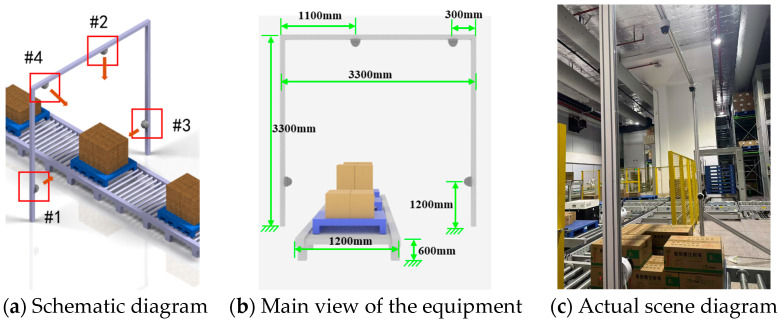
The equipment installation and data collection. #1–#4 are RGB cameras, with detailed installation specifications in Table 1.

In this work, we have constructed a multi-angle pallet image dataset from the conveyor line at an automated warehouse in the medical industry, which we designate as the GY Warehouse Box View 4-Dimension (GY-WSBW-4D) dataset. Specifically, the dataset comprises images of pallets in transport on the conveyor line, captured from four distinct angles. The image acquisition setup is illustrated in Figure 14. We developed automated scripts that interact with the warehouse’s electrical control system to achieve automatic positioning and capture during cargo transport. To ensure accurate relative positioning of the cargo, we implemented temporary stops at designated positions on the conveyor line during image capture, thereby avoiding position deviations caused by equipment movement and shooting delays. The dataset contains 3024 photographs in total. For each picture, we recorded the number of boxes, dimensions of each box, cargo name and specifications, and stacking pattern information, facilitating various research endeavors related to pallet information analysis. The equipment installation and data collection are shown in Figure 14 and Table 1.

Based on the capture conditions and stacking patterns, we annotated 1415 images with three categories of labels: ‘top’ labels for box upper surfaces, ‘front’ labels for front and side surfaces (originally labeled as ‘front’ and ‘flank’ but consolidated for processing), and ‘error’ labels for box surfaces in the environment that may affect the results.

Furthermore, due to the relatively rare occurrence of unstable stacking patterns in actual production (unstable stacks are typically adjusted manually before reaching the shooting point, though this does not imply the absence of stacking issues in actual production) and the lack of precise box position information (GY-WSBW-4D only contains stacking pattern and box shape information, without precise box position data), we established a supplementary dataset WSBW-Err containing unstable stacking patterns. This dataset includes approximately 150 images of pallets exhibiting tilting conditions and relative position information for each box. We utilize WSBW-Err for position detection algorithm experiments in Section 4.3. Table 2 summarizes the composition and distribution of our GY-WSBW-4D dataset for model training and evaluation.

### 4.2. Implementation Details

#### 4.2.1. Experimental Environment

All experiments used the PyTorch v1.8.0 framework on a workstation with an NVIDIA GeForce 4090 GPU (24 GB). We initialized the backbone network weights for the detection model training using a model pre-trained on the ImageNet dataset. The training process was configured with the following settings: 12 epochs for end-to-end training; AdamW optimizer with an initial learning rate of 0.0001, momentum of 0.9, and weight decay of 0.05. A WarmUp strategy was implemented, where the learning rate gradually increased to 0.9 during the first 1000 batch iterations, followed by a learning rate reduction at the 8th and 11th stages.

#### 4.2.2. Experimental Parameters

The implementation parameters were carefully tuned through extensive experimental validation across different processing stages:

- Image Preprocessing: The CLAHE algorithm was configured with a block size of 8 × 8 and clipLimit of 2.0. Gaussian filtering used a 5 × 5 kernel with σ = 1.0, while both morphological operations and median filtering employed appropriate kernel sizes (3 × 3 and 5 × 5, respectively).
- Edge Detection: The Canny operator was implemented with optimized thresholds (Th = 150, Tl = 75) and a 3 × 3 Sobel kernel size. These parameters were selected to balance edge preservation and noise suppression effectively.
- Line Detection: The Hough transform parameters were set as follows: ρ resolution of 1 pixel, θ resolution of π/180 radians, minimum votes of 50, minimum line segment length of 50 pixels, and maximum gap of 10 pixels. We employed a 45-degree classification threshold and a 5-degree merging angle threshold for line classification and merging.
- Corner Detection: The clustering distance threshold for corners was set to 10 pixels, with an angle tolerance of ±10 degrees. The minimum and maximum line segment lengths were defined as 0.1 and 0.5 times the ROI diagonal length, respectively.

#### 4.2.3. Model Architecture Details

For the comparative experiments in this study, we evaluated several detection models with different architectural designs. Table 3 provides an overview of the key components that constitute each model architecture, serving as a reference for understanding the subsequent experimental results.

#### 4.2.4. Evaluation Metrics

**Evaluation Metrics of Detection Network**

**Figure 15:**
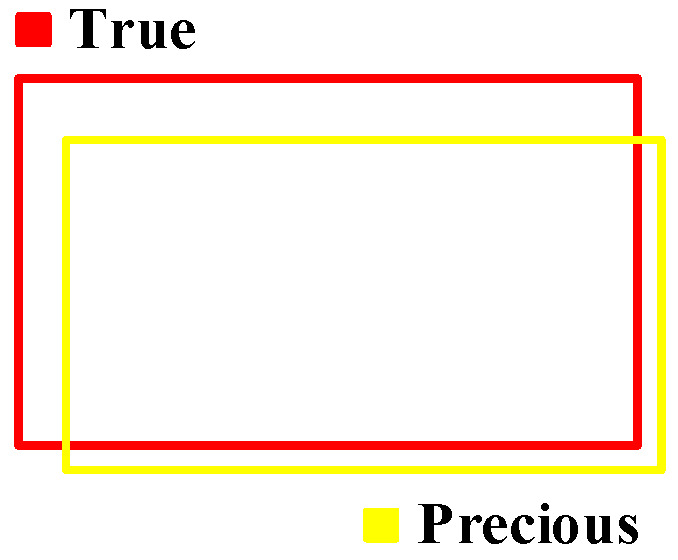
IoU value calculation.

The performance of the object detection network is evaluated using mean Average Precision (mAP). This metric is derived from the Intersection over Union (IoU), which quantifies the overlap between a predicted bounding box and its corresponding ground truth, as illustrated in Figure 15.

A predicted box is classified as a True-Positive (TP) if its IoU with a ground truth box exceeds 0.5. Conversely, it is considered a False-Positive (FP) if redundant or below this threshold for all ground truth boxes. A ground truth box undetected by any prediction is regarded as a False-Negative (FN). Based on these conditions, precision and recall are defined as:

(29) $$Precison=\frac{TP}{TP+FP}$$

(30) $$Recall=\frac{TP}{TP+FN}$$

The mAP is computed via the Precision–Recall (P-R) curve, generated by sorting predictions based on confidence scores and filtering by an IoU threshold of 0.5. The Average Precision (AP) is obtained as the area under this curve. Due to discontinuities, interpolation is applied at recall levels [0, 0.1, …, 1.0], with the maximum precision post each interpolation point used for area estimation. The achieved mAP is the mean AP across all object classes.

To quantitatively evaluate the performance of our proposed monocular image-based box pose estimation algorithm, we adopted a comprehensive evaluation metric system that assesses both position accuracy and pose accuracy.

**Position Accuracy Metrics**

Position accuracy is evaluated through multiple complementary metrics that capture estimation errors from different perspectives:

Mean Absolute Error (MAE): MAE measures the average magnitude of error between predicted vertex positions $\hat{p}$ and true positions p, calculated as:

(31) $$MAE=\frac{1}{N}\sum_{i=1}^{N}\left|{\hat{p}}_{i}-p_{i}\right|$$

where N is the number of vertices; this metric provides an intuitive understanding of coordinate measurement deviation, with lower values indicating better performance.

Root Mean Squared Error (RMSE): RMSE emphasizes more significant errors by computing the square root of the squared differences between predicted and actual positions:

(32) $$RMSE=\sqrt{\frac{1}{N}\sum_{i=1}^{N}{\left({\hat{p}}_{i}-p_{i}\right)}^{2}}$$

This metric is particularly sensitive to outliers, helping identify significant estimation errors.

**Pose Accuracy Metrics**

Pose accuracy is evaluated through metrics specifically examining angular relationships between vertices. Here, we use Relative Angular Error (RAE), which assesses the accuracy of estimated orientations by comparing angles between corresponding edges in predicted and true configurations:

(33) $$RAE=\frac{1}{M}\sum_{i=1}^{M}\left|{\hat{\theta }}_{i}-\theta_{i}\right|$$

where M is the number of measured angles, $\hat{\theta }$ represents predicted angles, and $\theta_{i}$ represents true angles.

**Accuracy Metrics**

We define Point Accuracy (PA) based on a position threshold $\tau_{p}$ and an orientation threshold $\tau_{\theta }$:

(34) $$PA=\frac{P_{ts}}{P}\times 100\%$$

where $P_{ts}$ is the number of vertices within the threshold range, and P is the total number of vertices. Expressed as a percentage, the Point Accuracy metric quantifies overall detection performance by combining both positional and orientational accuracy criteria into a single, intuitive measure. A prediction is considered successful if the position error is less than $\tau_{p}$ meters and the orientation error is less than $\tau_{\theta }$ degrees. Based on industrial application requirements, we set $\tau_{p}$ = 0.05 m and $\tau_{\theta }$ = 5°. This metric allows us to assess whether detected pallets exhibit unstable stacking conditions.

Furthermore, we define the Box Inclination Accuracy (BIA) to evaluate the algorithm’s ability to detect excessive carton tilting beyond the pallet edges. BIA is calculated by:

(35) $$BIA=\frac{Pc}{Pt}\times 100\%$$

where $P_{c}$ denotes the number of correctly predicted tilted cartons, and $P_{t}\;$represents the total number of detected tilted cartons.

#### 4.2.5. Camera Calibration Results

**Figure 16:**
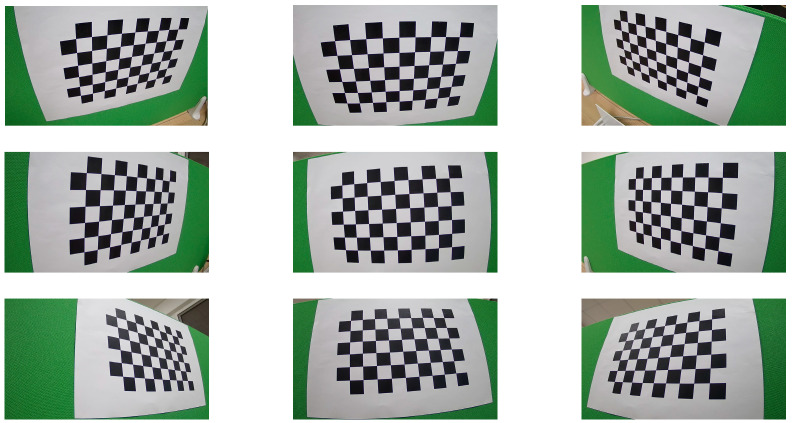
Schematic diagram of camera calibration process.

As shown in Figure 16, the study used an 8 × 6 checkerboard pattern for camera calibration, with each square measuring 20 mm. By adjusting the camera position for calibration purposes, nine images were captured from different angles.

The calibration was performed using OpenCV’s calibrateCamera method, with results shown in Table 4 and Table 5.

### 4.3. Training Process Analysis

To evaluate our proposed models’ training efficiency and convergence characteristics, we analyzed the training dynamics of both STEGNet and MixFEGNet during the optimization process.

**Figure 17:**
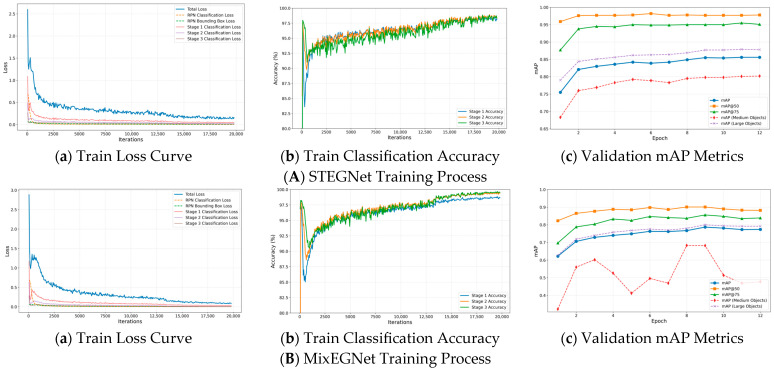
Train process curve.

Figure 17 illustrates the training dynamics of STEGNet and MixFEGNet, showing loss curves, accuracy progression, and mAP evolution throughout the training process. Both networks demonstrate stable convergence patterns, with STEGNet achieving lower final loss values and higher ultimate accuracy than the baseline. The loss curves reveal STEGNet’s three-phase learning process: rapid initial descent (0–1000 iterations), steady optimization (1000–3000 iterations), and final refinement (3000+ iterations). MixFEGNet exhibits similar loss reduction trends but converges slightly slower due to its architectural composition while achieving slightly lower final mAP on the GY-WSBW-4D dataset. These dynamics confirm that our models enhance detection precision while maintaining efficient convergence characteristics, with STEGNet optimizing for accuracy and MixFEGNet balancing performance with computational efficiency.

### 4.4. Comparative Experiments

To validate the effectiveness of our proposed detection network, we conducted comparative experiments on both the WSGID-B dataset (proposed by STCNet) and the GY-WSBW-4D dataset. We employed STCNet [60] as the baseline model for comparison with our proposed STEGNet and MixFEGNet. Additionally, we compared our proposed detection networks with several classic detection networks. Our experiments were implemented based on the MMDetection framework, with results shown in Table 6.

As shown in Table 6, we conducted comparative experiments on WSGID-B and GY-WSBW-4D datasets. On WSGID-B, our proposed STEGNet achieved 86.96% mAP, outperforming the baseline STCNet (86.13%), with notable improvements in front (+0.86%) and upper label (+0.77%) categories. On GY-WSBW-4D, STEGNet demonstrated even stronger results with 93.49% mAP (0.75% improvement over baseline), particularly excelling in upper-label detection (+1.3%). Our lightweight MixFEGNet maintained competitive performance despite size reduction, while traditional networks like FasterRCNN and CascadeRCNN showed stable but slightly inferior results. The more significant performance gaps in DETR Series and Yolox suggest that general-purpose architectures are less suitable for specialized warehouse cargo detection tasks.

**Figure 18:**
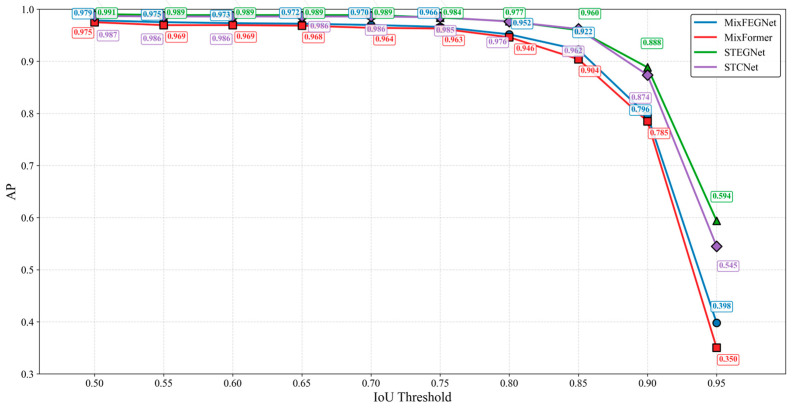
AP performance comparison.

To comprehensively evaluate the performance of the proposed models, we conducted a detailed comparative experimental analysis of MixFEGNet and STEGNet under different IoU thresholds. As shown in Figure 18, STEGNet demonstrates slightly better detection performance than the baseline model STCNet in the low-precision range (IoU 0.50–0.70). In the medium-precision range (IoU 0.75–0.85), STEGNet performs comparably to STCNet, demonstrating model stability. Notably, STEGNet shows significant advantages in the high-precision range (IoU 0.85–0.95). MixFEGNet consistently outperforms MixFormer-based networks across all precision levels. This indicates that our proposed EG-FPN module effectively enhances the model’s ability to localize targets precisely.

Table 7 shows comparative results on the WSBW-Err dataset, where our proposed STEGNet demonstrates superior performance with best-in-class metrics: MAE of 3.79 cm (3.3% improvement over STCNet), RMSE of 5.88 cm (19.0% reduction), and RAE of 1.5° (31.8% improvement). STEGNet achieves the highest detection accuracies with 63.27% PA and 80.8% BIA. While our lightweight MixFEGNet shows some performance trade-offs, with higher MAE (4.01 cm) and RMSE (8.13 cm), it still maintains acceptable detection accuracy (57.28% PA and 76.3% BIA) while significantly reducing computational demands, making it suitable for resource-constrained applications where balanced performance and efficiency are required.

### 4.5. Ablation Studies

To validate the performance of our proposed EG-FPN and verify the effectiveness of choosing Dynamic Head, we designed ablation experiments to evaluate the results, as shown in Table 8.

Ablation studies confirm the effectiveness of our architectural components. In STEGNet, Auto Fusion alone showed mixed results, but when combined with LSA, it achieved consistent improvements across all metrics (AP50: +0.5%, AP75: +0.2%, mAP: +0.5%). The complete configuration with Auto Fusion, LSA, and ECA achieved the best performance, with improvements of +0.7%, +0.2%, and +0.9% in AP50, AP75, and mAP, respectively. The impact was more pronounced in MixFEGNet, where LSA produced substantial gains (AP50: +2.4%, AP75: +2.7%, mAP: +4.4%), and the complete integration resulted in the most significant improvements (AP50: +2.5%, AP75: +3.9%, mAP: +4.9%). These results validate our architecture design and demonstrate the synergistic benefits of combining these modules in both network variants.

Table 9 presents ablation experiments evaluating the effectiveness of different components and algorithms in our edge detection pipeline. Our complete approach, which integrates preprocessing, Canny edge detection, edge filtering, and RANSAC-based line fitting, achieved the best overall performance, with an MAE of 2.86 pt, RMSE of 4.23 pt, and accuracy of 70.56%. The ablation results demonstrate the importance of each component: removing the preprocessing step led to significant performance degradation (MAE increased by 3.11 pt, accuracy dropped by 17.32%), while replacing Canny with Sobel edge detection resulted in reduced accuracy (63.17%) and higher error rates (MAE: 4.33 pt, RMSE: 8.47 pt). Notably, the absence of edge filtering showed the most severe impact on performance, with accuracy dropping to 45.52% and error rates more than doubling (MAE: 8.32 pt, RMSE: 16.74 pt). In line fitting methods, while both OLS and the Hough transform showed competitive performance (accuracies of 68.34% and 70.12%, respectively), our RANSAC-based approach demonstrated slightly superior results, particularly regarding error metrics.

### 4.6. Efficiency Analysis

Furthermore, we conducted evaluation experiments on model inference speed, model size, and complexity. The comparison results are presented in Table 10.

In our experimental evaluation, STEGNet demonstrated superior detection performance (mAP) on both datasets while maintaining significant advantages in model size (reduction of 5.29 MB), inference speed (increase of 0.13 FPS), and computational complexity (reduction of 5.73 GFLOPS) compared to the baseline STCNet. The significant inference speed improvement of MixFEGNet (21.28 FPS) compared to the baseline STCNet (12.67 FPS) and other transformer-based models like Sparse R-CNN (18.97 FPS) is primarily attributed to the strategic replacement of the Swin-T backbone with Mixformer architecture. Mixformer [61] integrates the strengths of CNN and Transformer through its efficient mixed attention mechanism, achieving a more favorable balance between global feature extraction capabilities and computational efficiency. By reducing computational complexity in the backbone, which typically accounts for the majority of model parameters and operations, MixFEGNet maintains robust detection performance while substantially reducing inference latency. It should be noted that MixFEGNet is designed explicitly for warehouse monitoring systems with GPU acceleration rather than CPU-only environments, as our preliminary tests indicate suboptimal performance (0.5–2 FPS) on mainstream CPUs.

### 4.7. Error Analysis

Therefore, this section analyzes the causes of errors and potential solutions. We studied error samples from both WSGD-Err and GY-WSGD-4D datasets and identified three categories of error sources. To ensure the engineering significance of our research algorithm, we automatically collected data (GY-WSGD-4D) in an operational automated warehouse, which reflects actual engineering application conditions. Among these, pallet stacking factors and environmental acquisition factors are issues we discovered in engineering applications.

#### 4.7.1. Limitations of the Proposed Method

Our proposed visual inspection algorithm, while effective in many scenarios, has several inherent limitations that define its operational boundaries:

- Color Dependency: The algorithm is primarily optimized for natural-color cardboard boxes (standard yellow–brown) commonly found in warehouse environments. The performance shows variations when processing different-colored boxes, with edge detection accuracy for low-contrast dark boxes decreasing by 4.95% points compared to natural-color boxes. High-contrast boxes also show a slight performance difference (1.31% point decrease in PA), as detailed in Section 4.7.2.
- Lighting Sensitivity: As quantified in our experiments, the algorithm’s performance varies under different lighting conditions. Edge detection accuracy decreases by approximately 7.53% points under dim lighting conditions and 10.57% points under uneven lighting compared to standard lighting conditions (see Section 4.7.3 for detailed analysis).
- Environmental Constraints: The algorithm assumes a relatively controlled warehouse environment. Background clutter (contributing to 13.7% of errors) significantly impacts performance, requiring additional preprocessing steps in complex real-world settings.
- Geometric Limitations: The current implementation struggles with high cargo stacks (3.4% of errors) and cases where box edges adhere to each other (6.8%), limiting its application in densely packed storage scenarios.
- Parameter Sensitivity: As shown in Table 11, approximately 26.5% of errors are related to algorithm parameter settings, indicating that the method requires careful calibration for each deployment environment.

Among these limitations, we have conducted further in-depth analysis on color variation robustness and lighting condition sensitivity, as these two factors significantly impact the practical application of our method in real-world warehouse environments and were explicitly highlighted in the evaluation process.

#### 4.7.2. Robustness for Cargo Box Color Variation

To systematically evaluate the algorithm’s performance across different cargo box colors, we selected test samples from the GY-WSBW-4D Test Set. The cargo boxes were categorized into three main color types:

1. **Natural-color boxes (as shown in Figure 11c,d):** standard yellow–brown cardboard boxes that represent the default color in most warehouse environments.
2. **High-contrast boxes (as shown in Figure 11a):** light-colored boxes (such as white) that create clear contrast with the floor and surroundings.
3. **Low-contrast boxes (as shown in Figure 11b):** dark-colored boxes that provide minimal contrast with shadows and dark surroundings.

Table 12 presents our target detection network (STEGNet) performance metrics and the edge detection algorithm across these different color categories.

Our analysis revealed several significant findings:

1. Target Detection Robustness: The STEGNet detection network demonstrated robustness across different box colors, with only a slight decrease (3.45%) in mAP for low-contrast boxes compared to natural-color boxes. This indicates that when properly trained, the deep learning-based detection component can effectively generalize across color variations.
2. Edge Detection Performance Variation: We observed interesting performance differences across box color categories using Point Accuracy (PA) as our evaluation metric (with thresholds $\tau_{p}$ = 5 pt). High-contrast boxes showed a slightly different accuracy (71.95% PA) than natural-color boxes (73.26% PA), possibly influenced by brightness variations and surface reflectivity characteristics. Low-contrast boxes presented a somewhat lower accuracy (68.31% PA), representing a 4.95 percentage point difference compared to natural-color boxes. This variation appears to be related to the reduced edge gradients in grayscale conversion, where dark surfaces create edge patterns that can be more challenging to distinguish, and shadow effects become more influential in the detection process.

In our future research, we plan to enhance our methodology by incorporating adaptive preprocessing based on detected box color, implementing multi-channel edge detection that better preserves color information and developing specialized enhancement techniques for low-contrast scenarios with dynamically adjusted detection parameters. These planned enhancements will further increase the algorithm’s versatility across diverse box colors in real-world warehouse environments, building upon the already promising foundation of our current approach.

#### 4.7.3. Sensitivity to Environmental Lighting Conditions

We conducted experiments under controlled lighting conditions using samples from our GY-WSBW-4D dataset to evaluate the impact of lighting variations on our algorithm’s performance. We established four distinct lighting scenarios that represent typical variations in warehouse environments:

1. Normal lighting: uniform overhead lighting (baseline condition).
2. Dim lighting: simulating early morning/evening warehouse conditions.
3. Bright lighting: simulating direct sunlight through windows.
4. Uneven lighting: normal overall illumination with strong directional light creating shadows.

Table 13 summarizes the performance impact of these lighting variations on both the target detection and edge detection components of our algorithm.

Our experiments revealed that lighting conditions significantly impact algorithm performance, with the edge detection component showing greater sensitivity than the target detection network. Under dim lighting conditions, edge detection accuracy decreased by 7.53 percentage points compared to customary conditions, as insufficient illumination led to loss of edge information during noise reduction and inconsistent edge strength measurement. The target detection component showed better resilience but experienced a 5.1% decrease in mAP under these conditions.

Bright lighting presented moderate challenges, with a 4.42 percent decrease in edge detection accuracy, primarily due to increased reflections on box surfaces and bright areas that affected edge details. The most challenging scenario was uneven lighting, which caused a 10.57 percentage point decrease in edge detection accuracy due to shadows creating false edges and inconsistent brightness levels across box surfaces.

In our future research, we plan to enhance our methodology by implementing adaptive thresholding techniques that adjust based on detected scene illumination, incorporating lighting normalization in the preprocessing stage, and exploring advanced deep learning architectures that can better handle illumination variations. We will also investigate hardware solutions such as controlled lighting installations or depth cameras that can provide illumination-invariant structural information. These planned enhancements will further increase the algorithm’s versatility across varying lighting conditions typically encountered in real-world warehouse environments, building upon the promising foundation of our current approach.

In conclusion, our error analysis reveals that the proposed algorithm faces several limitations that define its applicability boundaries and directions for future improvement. Based on our comprehensive analysis, we suggest the following targeted solutions for each identified limitation:

1. Color Dependency: Our experiments with natural-color, high-contrast, and low-contrast boxes (Section 4.7.2) demonstrated performance variations, particularly for edge detection on low-contrast boxes (4.95 percentage point decrease in PA compared to natural-color boxes). In our future research, we plan to implement adaptive color-space transformations before edge detection, develop color-aware algorithms that adjust parameters based on detected box color, and expand our training dataset to include more diverse box colors. These enhancements will improve the algorithm’s versatility across various cargo types in real warehouse settings.
2. Lighting Sensitivity: Our experiments across four lighting conditions (Section 4.7.3) revealed performance variations, especially under uneven lighting (10.57 percentage point decrease in edge detection accuracy compared to standard lighting). In future research, we plan to implement adaptive thresholding based on detected scene illumination, incorporate lighting normalization preprocessing, and explore depth camera integration to provide illumination-invariant structural information. From an engineering perspective, installing dedicated lighting systems in critical inspection areas would offer a cost-effective solution for maintaining consistent illumination.
3. Environmental Constraints: To overcome challenges caused by background clutter (13.7% of errors), we recommend enhancing our preprocessing algorithms with advanced background segmentation techniques and implementing context-aware filtering methods. Engineering solutions include installing uniform background panels in inspection areas and optimizing camera placement to minimize background interference, thus creating more controlled conditions without extensive facility modifications.
4. Geometric Limitations: For issues related to cargo stacking (tall stacks, edge adhesion, and occlusion accounting for 20.5% of errors), we will investigate multi-view fusion algorithms that combine information from different camera angles to resolve occlusion issues. Future work will also explore machine learning approaches (e.g., SVM or deep learning models) specifically trained to address edge adhesion problems. From an engineering perspective, adjusting the camera shooting distance and angle based on stack height can improve operational environments.
5. Parameter Sensitivity: To address parameter-related issues (26.5% of errors), we will develop an adaptive parameter tuning system that automatically adjusts threshold values based on detected environmental conditions and cargo characteristics. This will involve creating a comprehensive parameter optimization framework that considers the interdependencies between algorithm components. From an engineering standpoint, we will provide clear parameter calibration guidelines for different operational scenarios to ensure optimal performance across diverse warehouse environments.

By implementing these targeted improvements, we aim to enhance the robustness and applicability of our visual inspection algorithm across a broader range of real-world warehouse conditions, balancing algorithm sophistication with practical engineering considerations.

## 5. Conclusions and Discussion

This paper presents a vision-based method for detecting the stacked goods position in AS/RS, significantly contributing to automated warehouse management. The proposed approach demonstrates robust performance in real-world applications while offering several key advantages.

The innovative STEGNet architecture with EG-FPN balances computational efficiency and detection accuracy, achieving 93.49% mAP on the GY-WSBW-4D dataset. This performance improvement is particularly significant given the complex nature of warehouse environments. Alongside STEGNet, our lightweight MixFEGNet model achieved 89% mAP while significantly enhancing operational efficiency with the highest inference speed (21.28FPS, +68% over baseline) and smallest model size (186.19 MB, 66% of baseline), offering an excellent alternative for resource-constrained deployments, with only modest performance trade-offs. Moreover, integrating geometric constraints with edge detection algorithms provides a practical solution for accurate box position estimation, with position errors controlled within 4cm and rotation errors below 2°. Furthermore, developing the GY-WSBW-4D dataset contributes valuable resources for future research in warehouse automation.

However, our error analysis reveals several challenges that warrant further investigation. The current system’s performance is affected by three main factors: pallet stacking conditions (20.5% of errors), environmental variables (including lighting variations and cargo box color, 41.9% of errors), and algorithm parameters (26.5% of errors). Our experiments demonstrated that low-contrast boxes reduce edge detection accuracy by 4.95 percentage points compared to natural-color boxes, while uneven lighting conditions can decrease performance by up to 10.57 percentage points compared to standard conditions.

In terms of detection robustness enhancement, future work should focus on integrating depth information to improve edge detection accuracy and developing adaptive parameter adjustment mechanisms for varying lighting conditions and box colors. Implementing color-aware algorithms that adjust parameters based on detected box characteristics and adaptive thresholding techniques that respond to scene illumination would address key environmental limitations.

Regarding system optimization, research efforts should be directed toward investigating multi-sensor fusion techniques to address environmental limitations. Developing automated parameter optimization methods would significantly enhance system performance, while expanded dataset collection with diverse box colors and lighting conditions would improve the algorithm’s generalization capability across different warehouse environments.

For practical implementation considerations, future work should emphasize the design of standardized deployment guidelines for warehouse environments, including optimal lighting setup and camera positioning. The development of real-time monitoring and adjustment capabilities and seamless integration with existing warehouse management systems would further enhance the system’s practical utility.

These proposed improvements would further enhance the system’s reliability and practicality in industrial applications. The focus should remain on striking an optimal balance between algorithm sophistication and practical implementation requirements, ensuring high performance and cost-effectiveness in real-world deployments. Through continued research and development in these areas, the vision-based detection system can evolve to meet the growing demands of modern automated warehouse operations while maintaining robust performance under diverse operational conditions.

## Figures and Tables

**Table 1:** Camera installation.

Serial Number	Installation Location	Horizontal Shooting Angle	Vertical Shooting Angle	Dataset Name
#1	1200 mm from bottom to top on the left side of the shooting frame	Facing the center point of the largest stack of goods	Horizontal right	GY-WSBW-A
#2	The top of the shooting frame, facing the center of the cargo stack	Vertically downward	Vertically downward	GY-WSBW-B
#3	1200 mm from bottom to top on the right side of the shooting frame	Facing the center point of the largest stack of goods	Horizontal left	GY-WSBW-C
#4	Top of the shooting frame, 300 mm from right to left	Opposite to the right side of the conveyor line	Slant downward 45°∠	GY-WSBW-D

**Table 2:** Dataset composition and distribution.

Dataset Component	Total Images	Train Set	Val Set	Test Set	Annotations
GY-WSBW-A	756	248	53	53	‘top’, ‘front’, ‘error’
GY-WSBW-B	756	248	53	53	‘top’, ‘front’, ‘error’
GY-WSBW-C	756	248	53	53	‘top’, ‘front’, ‘error’
GY-WSBW-D	756	248	53	53	‘top’, ‘front’, ‘error’
GY-WSBW-4D	3024	991	212	212	1415 annotated images
WSBW-Err	150	105	22	23	Unstable stacking patterns

The dataset is split according to standard machine learning practices with a 70/15/15 ratio for training (Train Set), validation (Val Set), and testing (Test Set).

**Table 3:** Model architecture components.

Model	Year	Head	Backbone	Neck	Parameters
STCNet [60]	2022	Cascade	Swin-T	FPN	59.7 M
FasterRCNN [18]	2015	RPN + RoI	ResNet50	FPN	40.3 M
CascadeRCNN [19]	2018	Cascade	ResNet50	FPN	60.6 M
SparseRCNN [62]	2021	Learnable Proposal	ResNet50	FPN	38.7 M
Dino [14]	2022	DETR	Swin-T	Deformable	44.9 M
Co-DETR [15]	2023	DETR	Swin-T	Deformable	56.0 M
Yolox [12]	2021	YOLO Head	CSPDarknet	PANet	50.0 M
STEGNet	--	Dynamic	Swin-T	EG-FPN	49.7 M
MixFEGNet	--	Dynamic	Mixformer	EG-FPN	40.3 M

STCNet, STEGNet, Dino, and Co-DETR use Swin-Transformer Tiny (Swin-T) as their backbone architecture. FasterRCNN, CascadeRCNN, and SparseRCNN use ResNet50, while Yolox employs its specifically designed CSPDarknet backbone. MixFEGNet leverages the more efficient Mixformer (22 M) backbone. The EG-FPN neck in our proposed STEGNet and MixFEGNet enhances feature representation through gated fusion mechanisms and lightweight attention modules, providing more effective multi-scale feature modeling than traditional FPN while maintaining parameter efficiency. The significant parameter reduction in MixFEGNet is primarily achieved through combining the Mixformer backbone and an optimized EG-FPN implementation.

**Table 4:** Key parameters of camera intrinsic matrix used in experiments.

K	$f_{x}$	$f_{y}$	$c_{x}$	$c_{y}$
px	1.23 × 10^3^	9.35 × 10^2^	1.22 × 10^3^	5.22 × 10^2^

**Table 5:** Camera distortion coefficients used in experiments.

d	$k_{1}$	$k_{2}$	$p_{1}$	$p_{2}$	$k_{3}$
	−3.67 × 10^−1^	2.05 × 10^−1^	−1.18 × 10^−3^	1.14 × 10^−4^	−1.30 × 10^−1^

**Table 6:** Comparative experimental results based on WSGID-B and GY-WSBW-4D.

Dataset	WSGID-B [60]			GY-WSBW-4D		
mAP	Front	Upper	Total	Front	Upper	Total
STCNet [60] (Baseline)	**92.80**	**72.80**	**8** **6 .** **13**	**92.30**	**93.45**	**92.74**
FasterRCNN [18]	91.73	71.82	85.09	91.64	92.47	91.96
	(1.07)	(0.98)	(1.04)	(0.66)	(0.98)	(0.78)
CascadeRCNN [19]	93.01	72.31	86.10	92.55	93.03	92.73
	( 0.21 )	(0.49)	(0.02)	(0.25)	(0.42)	(0.01)
SparseRCNN [62]	90.84	68.98	83.49	90.45	90.88	90.61
	( 1.96 )	(3 .82 )	(2. 64 )	(1. 85 )	(2. 57 )	( 2 . 13 )
Dino [14]	91.14	69.38	83.88	90.88	91.24	91.02
	(1.66)	(3.42)	(2.25)	(1.42)	(2.21)	(1.72)
Co-DETR [15]	93.18	72.52	86.24	92.68	93.28	92.86
	( 0. 38)	( 0. 28)	( 0. 11)	(0. 38 )	( 0 . 17 )	(0. 15 )
Yolox [12]	90.06	67.33	82.47	89.87	90.34	90.05
	(2.74)	(5.47)	(3.65)	(2.43)	(3.11)	(2.69)
MixFEGNet	92.38	72.25	85.66	87.60	91.37	89.03
	( 0.42 )	( 0.55 )	( 0.46 )	(4.7 0 )	(2.08)	(3.71)
STEGNet	93.66	73.57	86.96	**92.72**	**94.75**	**93.49**
	( 0.86 )	( 0.77 )	( 0.83 )	(0.42)	(1.3)	(0.75)

The items highlighted in **bold** are the best performance values;** grey** are the baseline values. Values in parentheses indicate improvement or decrease compared to the baseline, with red numbers showing improvement and green numbers showing decrease. The complete architectural details of FasterRCNN [18], CascadeRCNN [19], SparseRCNN [62], Dino [14], Co-DETR [15], and Yolox [12] are presented in Section 4.2.3, Table 3, while STCNet was configured according to the state-of-the-art parameters established in Study [60].

**Table 7:** Comparative experiments of Box Edge Detection Algorithm experiments on the WSBW-Err dataset.

Models	MAE (cm)	RMSE (cm)	RAE (°)	PA (%)	BIA (%)
STCNet [60]	3.92	7.26	2.2	60.34	78.2
(baseline)					
MixFEGNet	4.01	8.13	2.6	57.28	76.3
	(0. 09 )	(0.87)	( 0 .4)	(3.06)	(1.9)
STEGNet	3.79	5.88	1.5	63.27	80.8
	(0.13)	(1.38)	(0.7)	(2.93)	(2.6)

Values in parentheses represent differences from baseline, with red/green indicating improvements/degradations. For missing detection points (not detected or * out of the detection range by more than 10 cm), when calculating MAE and RMSE, the error values are set to the point accuracy threshold (5 cm). Rotation angle deviation (RAE) calculations exclude missing detection points, as rotation angle errors are not related to model detection performance but to the accuracy of orientation estimation for successfully detected objects. * Errors in the edge detection algorithm usually cause points exceeding the detection range by more than 10 cm and can be classified as undetected.

**Table 8:** Ablation study results of STEGNet and MixFEGNet on GY-WSBW-4D dataset.

Auto Fusion	LSA	ECA	STEGNet			MixFEGNet		
			AP50	AP75	mAP	AP50	AP75	mAP
			**87.8**	**83.8**	**78.6**	**85.7**	**80.0**	**72.5**
**√**			88.2	83.1	78.4	86.3	79.9	72.4
			(+0.4)	( − 0.7)	( − 0.2)	(+0.6)	( − 0.1)	( − 0.1)
**√**	**√**		88.3	84.0	79.1	88.1	82.7	76.9
			(+0.5)	(+0.2)	(+0.5)	(+2.4)	(+2.7)	(+4.4)
**√**		**√**	88.3	83.8	78.9	88.2	81.2	75.3
			(+0.5)	(0.0)	(+0.3)	(+2.5)	(+1.2)	(+2.8)
**√**	**√**	**√**	**88.5**	**84.0**	**79.5**	**88.2**	**83.9**	**77.4**
			(+0.7)	(+0.2)	(+0.9)	(+2.5)	(+3.9)	(+4.9)

The baseline for STEGNet is STCNet, and the baseline for MixFEGNet is the MixFormer-based detection network. The items highlighted in **grey** are the baseline values. Values in parentheses represent differences from baseline, with red/green indicating improvements/degradations.

**Table 9:** Ablation experiments for the Pallet Edge Detection Algorithm.

Algorithm				MAE (pt)	RMSE (pt)	PA (%)
Pre- Processing	Edge Detection	Edge Filtering	Line Fitting			
**√**	**Canny**	**√**	**RANSAC**	**2.86**	**4.23**	**70.56**
	Canny	√	RANSAC	5.97	10.49	53.24
√	Sobel	√	RANSAC	4.33	8.47	63.17
√	Canny		RANSAC	8.32	16.74	45.52
√	Canny	√	OLS	3.13	5.01	68.34
√	Canny	√	Houph	2.99	4.33	70.12

When calculating MAE and RMSE for missing detection points, the error values are set to the point accuracy threshold (5 pt). Our method is shown in **Bold**. MAE and RMSE are calculated based on 2D coordinates before 3D pose estimation.

**Table 10:** Evaluation of model inference speed, size, and complexity.

Model	Speed (FPS)	Model Size (MB)	GFLOPS
STCNet [60]	**12.67**	**280.79**	**130.32**
(baseline)			
FasterRCNN [18]	15.50	235.31	110.07
	(2.83)	(45.48)	(20.25)
CascadeRCNN [19]	10.20	269.43	121.32
	(2.47)	(11.36)	(9.00)
SparseRCNN [62]	18.23	238.80	115.47
	(5.56)	(40.48)	(14.85)
Dino [14]	8.31	420.11	181.73
	(4.36)	(139.32)	(51.41)
Co-DETR [15]	9.18	396.50	165.35
	(3.49)	(115.71)	(35.03)
Yolox [12]	18.97	209.83	119.45
	(6.3)	(70.96)	(10.87)
STEGNet	12.80	275.50	124.59
	(0.13)	(5.29)	(5.73)
MixFEGNet	21.28	186.19	113.68
	(9.15)	(94.6)	(16.64)

The items highlighted in **grey** are the baseline values. Values in parentheses represent differences from baseline, with red/green indicating improvements/degradations.

**Table 11:** Error analysis.

Main Reason	Details	Frequency	Error Precent
Stacking factors	Super-high cargo stack	4	3.4
	Cargo box edge sticking	8	6.8
	Cargo box surface texture	13	11.1
	Cargo box cover	12	10.3
Collection environmental factors	Background confusion	16	13.7
	Reflective surface of cargo box	18	15.4
	The surface of the cargo box is too dark	6	5.1
	Shadow	9	7.7
Algorithm parameter factors	Confidence threshold is too high or too low	14	12.0
	The preprocessing algorithm parameters are too large or too small	8	6.8
	Edge filtering threshold is too large or too small	9	7.7

Based on accuracy experiments, we found that the algorithm still has an error rate of approximately 15–30% on the WSGD-Err dataset.

**Table 12:** Performance analysis across different box colors.

Box Color Category	STEGNet Detection (mAP)	Edge Detection Accuracy (PA)
Natural-color	94.87%	73.26%
High-contrast	93.21%	71.95%
Low-contrast	91.42%	68.31%

Each color category contains 50 test images specifically selected from 212 test images and 1609 additional images not used during training in the GY-WSBW-4D dataset, for 150 test samples.

**Table 13:** Performance analysis under different lighting conditions.

Box Color Category	STEGNet Detection (mAP)	Edge Detection Accuracy (PA)
Standard lighting	93.49%	71.85%
Low lighting	88.73%	64.32%
Bright lighting	90.12%	67.43%
Uneven lighting	86.45%	61.28%

Each lighting condition test used 40 images specifically selected from the 212 test images and 1609 additional images not used during training in the GY-WSBW-4D dataset, for 160 test samples.

## Data Availability

All the data supporting the findings of this study can be made available upon reasonable request.
